# Experiences of patients who developed oral mucositis during solid neoplasms treatment: a Ugandan qualitative study

**DOI:** 10.1186/s41687-021-00301-5

**Published:** 2021-03-06

**Authors:** Adriane Kamulegeya, Damalie Nakanjako, Jackson Orem, Harriet Mayanja-Kizza

**Affiliations:** 1 Department of Dentistry, Oral maxillofacial unit, School of Health Sciences, Makerere University College of Health Sciences, Kampala, Uganda; 2 Department of Medicine, School of Medicine, Makerere University College of Health Sciences, Kampala, Uganda; 3 Uganda Cancer Institute, Kampala, Uganda

**Keywords:** Oral mucositis, Chemotherapy side effects, Cancer

## Abstract

**Background:**

Research on the management of complications of chemotherapy is important in facilitating the growing approaches to individualized patient management. Hence the need to document patient’s perspectives about chemotherapy-induced mucositis and the support they need from cancer care teams.

**Methods:**

We carried out a qualitative study using in-depth interviews (IDI) and focus group discussions (FGD). We collected patient’s experiences on chemotherapy-induced mucositis by conducting 5 FGD and 13 IDIs.

**Results:**

One glaring improvement that we need to make is the provision of information and counseling before, during, and after chemotherapy. Additionally, we need to explore inexpensive mucositis preventive strategies to aid our patients as they undergo treatment.

**Conclusion:**

As a country, we must move away from taking cancer patients’ needs as those of common tropical diseases. This will allow us to provide that extra help needed outside the usual diagnosis and administration of medication.

## Background

Patients undergoing chemotherapy experience different levels of complications ranging from nausea, vomiting, diarrhea, loss of appetite, alopecia, gastrointestinal mucositis to mention but a few [1]. Therefore research on these complications and how to mitigate them in different populations is vital. Although oral mucositis depends on patient factors, regimen, and steps taken during administration, it is still a common side effect and major chemotherapeutic limiting complication [2]. Unfortunately, very few studies focusing on patients’ experiences of mucositis have been carried out in developing countries more so in Sub Saharan Africa [3].

Mucositis by way of definition is an inflammatory process of the oral and/or gastrointestinal tract caused by high-dose oncology chemotherapeutic agents. Digestive tract mucositis is a result of mucosal injury across the continuum of oral and gastrointestinal mucosa i.e. from the mouth to the anus [4].

The incidence and severity of mucositis among patients undergoing chemotherapy is widely variable. It depends on medicines and dosage used, how it’s administered, preventive steps taken, and how the mucositis is evaluated [5]. Although several interventions such as slow infusions, use of ice chips, palifermin, and photoiomodulation therapy have been shown to be effective against mucositis [6], their use is not widespread in developing countries*.* Studies have shown incidences ranging from 40% to greater than 70% [7, 8]. A study from South Africa that used patients self-reported mucositis put the figure at nearly 72% [3]. Therefore mucositis is an important chemotherapy side-effect that needs to be explored in our Ugandan setting. Although Uganda as a country is a leader in palliative care especially when it comes to the provision of morphine in the management of pain [9], mucositis prevention, oral health, and dietary management among cancer patients are still wanting.

The Uganda Cancer Institute (UCI) being one of the oldest oncology centers of excellence in Africa [10], needs to set the pace on supportive care for cancer patients in Africa. It is only through area-specific studies that we will be able to develop evidence-based knowledge in oncological care delivery. Research that explores patients’ experiences of radiotherapy and or chemotherapeutic treatment in a low-income country like Uganda, would provide many learning opportunities to other developing nations. The results will be a source of knowledge on patients’ information, dietary and oral hygiene counseling needs. The study will provide us with the special requirements that must be addressed before our patients start chemotherapy.

Although some studies from East Africa describing patients’ experiences when undergoing chemotherapy have been published [11, 12], none of them focused on oral mucositis. On the other hand, globally patient-reported outcomes measures have increasingly taken center stage [13], and as such mucositis has not been left behind. The effect of mucositis on the different aspects of the quality of life has been well documented albeit to varying degrees [14]. However, studies with a qualitative angle are still few and in most cases have insufficient power. This makes it difficult to carry out more authoritative systematic reviews [15, 16]. Given the fact that oral mucositis a continuum of gastro-intestinal mucositis is a dose /schedule modifying complication of chemotherapy [17], there is a need to identify areas of interventions that can reduce the suffering among patients undergoing cancer treatment. To capture what it means and the potential interventions, for Ugandan patients, a qualitative approach was chosen. This aimed at describing the experiences of patients who have had oral mucositis following chemotherapeutic treatment.

## Methods

The study was conducted at the outpatients’ department of Uganda Cancer Institute a national oncology center in Uganda between 2018 and 2019. Uganda Cancer Institute (UCI) is an 80-bed public health facility that specializes in handling cancers in Uganda. It has regional centers in Mbarara, Mayuge, and Arua that are already running with two more expected on Board. Unfortunately, like many developing countries staffing shortages are acute with the country having only 20 oncology specialists as per the Ministry of Health website as of December 2020. The study was approved by the UCI Research and Ethics Committee (UCI REC REF: 13–2016) and Makerere University School of Health Sciences REC under a broader study on mucositis. Adult cancer patients diagnosed with a solid malignancy who had experienced oral wounds /ulcers following chemotherapy were invited to participate. All participants received general information about the research. A full written consent procedure was embarked on for those who agreed to be enrolled. The participants were recruited by an experienced oncology nurse but data collection was done by the principal investigator. Those recruited were patients who had had chemotherapy within the previous 6 months. The other inclusion criterias were ability to stand the interview and to speak either Luganda or English. All those who were too sick to stand the interview were excluded. The reason we chose subjects with self-reported wounds/ulcers following chemotherapy was that it has been shown to closely reflect the actual clinical picture of mucositis [18]. We, therefore, felt they would be “information and experience-rich”. Patients were excluded from the study if the interview would interfere with their turn to see the oncologist or getting their visit dose.

Data was collected through separate in-depth interviews (IDIs) and focused group discussions (FGDs) using semi-structured qualitative interviews with 13 cancer patients and 5 FGDs. The qualitative interview allowed a relatively free flow of information. This led to a detailed description of oral mucositis individual experiences. An interview guide consisting of open-ended questions with inbuilt targeted probes was used for each interview. Two of the questions in the interview schedule were “Can you tell me about your experience of being treated with chemotherapy right from the time you got the first treatment?” and “Please share with me the most disturbing effects of the cancer medication that you have experienced Probe if not mentioned: (What about ulcers in the mouth) Did you get any problems in the mouth?).”

The IDIs lasted anywhere between 15 to 40 min, the participants who had fewer disturbances from the mucositis gave shorter interviews. We used the first seven interviews as the start of the analysis and had decided apriori that five more interviews following saturation would be conducted before ending the IDIs. The FGDs lasted between 60 to 100 min. Each FGD was language homogenous i.e. either Luganda or English. A group consisted of 5–7 individuals each and they were heterogeneous for sex and age. The number of FGDs was predetermined at 5 each not exceeding seven participants as a budgetary decision. Interviews took place in one of the consultation rooms at the UCI outpatients department (OPD). Interviews were audio-recorded. Field notes were also made to help capture the body language of the individual during the interviews.

Both in-depth interviews (IDIs) and focus group discussions (FGDs) were done on purposively selected patients. In Addition to seeking out-patients who met our set criteria, for the FGDs we had to ensure that there was language homogeneity in each group. The choice of using both IDIs and FGDs was to try and boost the strength of each by way of triangulating the information gained to increase the validity and reliability of the study [19]. The two approaches were three months apart and an effort was made to ensure that FGD participants had not participated in the initial IDIs.

We used IDIs and FGDs in our study gaining from the strength of each. Whilst IDIs gave more privacy and hence more freedom to air out their experiences, we found out that FGDs provided an opportunity for participants to collaborate their experiences. The interjections and discussions led to group consensus and advice on some of the issues raised by a member.

This was a qualitative, descriptive, exploratory design [20]. Data collection and analysis took place simultaneously. Interviews and field notes were transcribed verbatim and then analyzed using inductive content analysis [21]. The content analysis included assigning the same codes to similar meaning (e.g. salty water and warm water with salt). Coding was done by two people then followed by code revision, harmonization, and aggregation to ensure that similar units of information had the same code leading to thematic areas. This process was done using atlas ti scientific software development GMBH Berlin Germany.

The information from the IDIs and the FGDs was examined and the categories were grouped into subthemes thus allowing the main themes to evolve from the data instead of imposing a predetermined framework. No new themes emerged from the analysis of the FGDs group interview. The authors agreed on the analysis of the initial codes and themes.

We emphasized the capture of patients’ own words to keep the authenticity and credibility of the study. By using FGDs, it gave us a chance to compare with IDIs hence increasing the reliability and validity of our results.

We compared different IDIs and FGDs but also compared IDIs to FGDs. Data collection and analysis were considered complete when no new themes were generated.

Each participant received a $5.5 transport refund as appreciation for participation in the study.

## Results

The mean age of the IDIs participants was 41.1 years (SD 4.0), of whom 5 (38.5%) were male. By the seventh interview, we felt we had saturation. We went ahead and added on the five and we were satisfied that there was no new information emerging. However, we carried on with a thirteenth interview since he was male yet we had few male participants. The mean age of the FGDs was 34.5 years (SD 12.4), with 15 (53.6%) females. The highest number of participants were breast cancer patients (20.0%) followed by choriocarcinoma, at 12.5%. Among the male participants, the highest number had Kaposi’s sarcoma. Nearly all our patients were on multi-agent chemotherapy, 17.5% treated with cyclophosphamide, Adriamycin, and 5 fluoro-uracil (CAF). This was followed by a combination of etoposide, methotrexate, actinomycin D, cyclophosphamide, and vincristine (EMACO) and only two participants were on paclitaxel as a single drug regimen (Table 1).

**Table 1:** Participants descriptive statistics

		IDIs	FGDs
Age			
Gender	Female	8	15
	Male	5	13
Cancer diagnosis	Breast cancer	8	4
	Chorio carcinoma		5
	Kaposi’s Sarcoma	2	1
	Ovarian Carcinoma		2
	Cervical Carcinoma		2
	Oesophageal carcinoma		2
	Colorectal carcinoma	1	1
	Pancreatic carcinoma		1
	Small cell lung carcinoma	1	1
	Nasopahrgeal carcinoma		1
	Sinonaso carcinoma	2	1
	Osteosarcoma		2
	Rhabdosarcoma		1
	Fibrosarcoma		4
Chemotherapy	Cyclophosphamide, Adriamycin 5 fluoro-uracil (CAF)	8	4
	Etoposide, Methotrexate, Actinomycin D, Cyclophosphamide Vincristine (EMACO)		5
	Bleomycin and Vinicristine (BV)	2	1
	5 fluoro-uracil, Leucovorin and Oxaliplatin (FolFox-6)	1	
	Cisplatin and Etoposide	1	
	Gemicitabine, Bleomycin, Etoposide and Cisplatin		1
	Cisplatin and Etoposide	1	
	Cisplatin and 5 Fluoro Uracil		2
	Cisplatin and Paclitaxel		3
	Doxorubicin, Vincristine, Cytarabine and Dounorubicin		1
	Bleomycin, Vinicristine and Paclitaxel		2
	Ifosphamide and Doxorubicin		1
	Bleomycin, Etoposide and Cisplatin		1
	Cisplatin and Doxorubicin		2
	Oxaliplatin and Capecitabine		1
	Vincristine,Adriamycin and Dacabazine		1
	Gemicitabine and Docetaxel		1
	Cyclophosphamide,Adriamycin, Vincristine and Prednisolone (CHOP)	1	
	Paclitaxel		2

Five themes, which cut across several categories, were constructed. Table 2 illustrate the challenges faced by cancer patient due to oral mucositis (OM).

**Table 2:** shows the categories and thematic map from the inductive analysis

Theme 1 Pre chemotherapeutic information and counselling	Theme 2 Symptoms experienced	Theme 3 The horror of eating	Theme 4: Oral care challenges	Theme 5: Needs
Access to counselling	Worst side effect and its consequences	Counselling on eating	Oral care experience	Education and Psychosocial needs
Provider of information	Timing of oral ulcers	Tolerable foods	Armamentarium used for oral care	Compassionate care
Quality of information got	Control of ulcers and resultant pain	Access to tolerable foods	Effectiveness of oral care method	Improved access to medicines

### Theme 1 pre chemotherapeutic information and counseling

Information and counseling are very vital for patients after cancer diagnosis and throughout their treatment journey. All participants talked about the need to be prepared for the side effects of chemotherapy. These were categorized into three; access to counseling, information provider, and quality of the information.

#### Access to counseling

Most participants didn’t have a chance at pre-chemotherapeutic counseling. Information was many a time got from other patients while waiting for treatment. This was well demonstrated in the level of agreement among participants in the first FGD:

1. *FGD1 P 1 “Actually they don’t warn you it is only after you ask. When the ulcers start then they tell you. ‘ha that’s is expected.’ But I had heard about them from patients so I googled and that’s how I came to learn more about the oral sores.”*
2. *FGD1 P 2 “I was told about hair loss and vomiting.”*
3. *FGD1 P 3 “I wasn’t told anything so I didn’t know. They just gave me medicine. The counselor wasn’t around that day so I was not counseled.”*
4. *FGD1 P 4: “I wasn’t told but I read.”*
5. *FGD1 P 5; “I wasn’t told but only got wounds.”*
6. *FGD1 P 6; “I was told about vomiting.”*
7. *FGD1 P 7: “As for me the doctor was called to attend to someone else and probably that distracted him from telling me. When I came back and complained about the ulcers then I was told that they are expected.”*

#### Information provider

Many participants expressed the desire to get sufficient time with the health care providers so that they can ask questions. Those who had a nurse or doctor give them information was very appreciative and felt confident even when the side effects were horrible. The following statements reflect a participant’s view: *“A very kind gentle nurse told me about hair loss, vomiting and oral sores so I was never surprised.*” (IDI 6).

#### Quality of information got

Those who got information from none health personnel, had semi-accurate, scary to out-rightly misleading information such as *“When the counselor started the session, she told me that the medicine was poisonous. So I asked her why then give it to us. She then asked me to leave and she talked to my daughter.”(IDI 11).*

### Theme 2 symptoms experienced

Oral sores (ulcers), diarrhea, vomiting, loss of appetite, fatigue, loss of hair, and general weakness were among the symptoms reported by most participants. These symptoms affected their daily activities and social lives. The 4th FGD expressed their experiences as follows:

1. *FGD4 P 1; “The first chemotherapy dose caused a loss of appetite and I became very thin. I was not knowledgeable. Diarrhea was the worst.”*
2. *FGD4 P 2; “oral sores were horrible I had the appetite but I couldn’t eat.”*
3. *FGD4 P 3; “Running stomach and loss of appetite were a nightmare.”*
4. *FGD4 P 4; “Difficulty in speech not because of the pain but rather heaviness of the tongue.”*
5. *FGD4 P 5; “Vomiting was really bad”*
6. *FGD4 P 6; “loss of my capacity to have sex with my wife. She started feeling rejected.” All discussants said they lost the sexual urge.*
7. *FGD4 P 7; “My menstrual cycle ceased up to date.”* A lady interjected
8. *FGD4 P 5 “So I may never be able to have children? “*Asked another group member
9. *FGD4 P 7 “Sores and constipation were my worst.”*

### Worst side effect and its consequences

The resultant pain due to oral mucositis made eating and speaking very difficult and in some instances led to the interruption of treatment. Pain reduced the patients’ urge to take in most food stuff. Thus leading to extremely low energy levels. Additionally, the patients dreaded speaking either on the phone or face to face. This greatly affected their social lives. Some participants’ oral ulcers were the tip of the iceberg since they had sores in the anal site as well. These quotations illustrate the patient’s suffering:

*“Ulcers and the accompanying pain were my worst. They affected me in the mouth, private parts, and even the area of fecal outlet. Hey hey, I worry about them. I even had to delay one of the cycles due to ulcers” (IDI 5)*

*“I lost a lot of weight due to failure to eat because of the sores. So I resorted to drinking as much as I could.” (IDI 7)*

### Timing of oral sores/ulcers

The timing of onset varied from those who got ulcers a day or two after the first chemotherapeutic administration to those who had them on the second or third cycle. The degree of ulceration differed for the same patient during different cycles. These sentiments can best be captured in the quotes below:

*“The first time I got them was after the second cycle but they ceased on the fourth cycle. They were worse with each cycle. I have had two different regimens and the second time around, I got sores on the first cycle. Those were more intense on the second and third cycle.” (IDI 12)*

*“Two days after the first cycle I got sores which healed about 4 days down the road. I had them just that one time.” (IDI 6)*

### Control of ulcers and resultant pain

Participants used an array of interventions to mitigate the pain but also promote quick healing of the sores. Some were medically prescribed while others were guided by community knowledge. Many participants used a cocktail of things in a desperate move to get rid of the sores. The options that were used are best captured by the following statements from the 1st FGD and 10th IDI.

1. *FGD 1 P 1 “I used BBC spray (Amoun pharmaceutical Egypt) and fluconazole, Sodium bicarbonate rinses to avoid infection. The sores didn’t grow bigger. I also had antibiotics. I used Kamunye (Hoslundia opposita) rinses and it helped”.*
2. *FGD 1 P 2 “Sere (Bidens pilosa) I chewed and also rinsed just the way we used to do it during the old days when we got injured while digging. Just three days the ulcers were gone.”*
3. *FGD 1 P 3 “Omwetango (chenopodiaceae) with salt but the ulcers around the teeth are still a problem.”*
4. *FGD 1 P 4 “I used salty rinses.”*
5. *FGD 1 P 5 “weren’t that bad so I didn’t use any medicine”*
6. *FGD 1 P 6 “Didn’t use anything but recently they gave me ora-cure gel (Amoun pharmaceutical Egypt) that I apply before I eat.”*

*“Leucovorin was helpful when I take it the wounds get better. When I take it immediately after treatment, I don’t get sores but even if I delay by a few minutes they start developing. I was given a tube blue and white in color codragel something that would numb them even down through. If I have no money for leucovorin I put off chemotherapy till I have the money to avoid wounds. I was given fluconazole but it didn’t help.” (IDI 10)*

### Theme 3 the horror of eating

The patient had several challenges in terms of feeding and we arrived at three major categories as shown in Table 2.

#### Counseling on feeding

Unfortunately, most of the participants didn’t get information about food stuff that are gentle on the sores during eating. So they had to figure it out as they went through that phase of treatment.

*“Yes I was told about the oral sores but not what I could do to relieve them or foods that I could take when I got them.” (IDI 5)*

*“I saw patients with these wounds. A nurse told me about use of ice-cold water after I developed the sores.” (IDI 9)*

#### Tolerable foods

Most participants had serious challenges in food items that were tolerable and many had to give up on their favorite food stuff just to keep the pain away. Some of the choices taken are captured in the patients’ sentiments expressed below:

*“Tried Yogurt and juice but it worsened the pain. Honey was the worst I never tried it again. I would try a small piece of pumpkin but even tears would drop. Watermelon was most favorable.” (IDI 8)*

*“Was taking cocktail juice, millet porridge. and water. I avoided salty foods. As much as I like ice I couldn’t try. Although I had no appetite, the wounds made it worse.” (IDI 7)*

*“Was only able to eat stiff porridge and beans the rest I didn’t want. Was able to take pawspaws but anything chewy I din’t want. Even beans I would only take the soup. I would close my eyes to eat.” (IDI 2)*

#### Access to tolerable foods

Although some participants had challenges in getting food items that were gentler on the oral sores, many had family support that helped avail items that they could feed on. This is shown in the comments below:

*“Well unfortunately the money that is left at home is a constant and you have to feed the children so you can’t afford the items that may be as comfortable.” (IDI 13)*

*“Mum helped a lot especially by making juices that were comfortable to take.” (IDI 9)*

### Theme 4: Oral care challenges

#### Oral care experience

There was variation in the experiences as expressed by the participants probably in line with the level of mucositis that they had. The excerpts below give an idea of what the participants went through during cleaning their mouths.

*“Ha?!! Can you brush your mouth when you have sores? It was very hard especially the first one week. I used water to rinse the mouth. This was the same on the second cycle.” (IDI 1)*

*“Wow brushing? You wouldn’t want a toothbrush to touch your mouth. But I had to feel fresh so I would try. Ora-cure gel helped. I would apply it every after brushing to soothe the pain.” (IDI 2)*

*“Hmm Hmm couldn’t brush but mother gave me warm water with salt and I would rinse. On spitting after rinsing I would see the chaff come out.” (IDI 9)*

#### Armamentarium used for oral care

Participants tried tooth brushing, using chewing sticks, gauze with plain water as a way of keeping their oral cavity clean but avoiding exacerbating the pain from the oral sores. This was captured by the comments below from FGD 2.

1. *FGD 2 P 1 “I used chewing sticks for brushing because they are gentle and not as painful. It has become my routine since then.”*
2. *FGD2 P 2 “I would pull it out and clean with a toothbrush because it has a coating in the morning. She illustrated how she does it by pulling out the tongue.”*
3. *FGD 2 P 3 “Attendant she can’t allow us to touch her mouth we can’t even get her to rinse.”*
4. *FGD 2 P 4 “I was able to brush gently with a tooth brush”*
5. *FGD2 P 5 “I couldn’t brush neither rinse. Tried oral salty rinses ounce and I failed so I gave up.”*

#### Effectiveness of oral care method

Participants tried different ways of keeping their oral cavity clean but those methods didn’t confer to them the level of hygiene they were used to. This was illustrated in the patients’ comments below:

*“Initially I couldn’t clean but four days after I got something sent by my sister from the UK that I would use with gauze. Sometimes I would try salty rinses. I would feel clean for a very short time.”(IDI 5)*

*“Tried brushing gently and rinsing with lukewarm salty water but it was inadequate. I felt a bit of bad odor in the mouth.” (IDI 7)*

### Theme 5: needs

The patient needs varied from general needs related to cancer management to those specific for oral sores. However, we felt the three categories as shown in Table 2 captured this research aspect well enough.

#### Education and psychosocial needs

*“Counselors and more medical staff are needed. Many patients run away when we are seated due to the scary things they hear from others. Cancer patients need a lot of time to be counseled.” (IDI 7)*

*“Health personnel should take time and talk to those who have the ulcers tell them what kind of foods cause the least discomfort.” (IDI 2)*

#### Compassionate care

Although patient acknowledged the limited human personnel to serve the unique needs of cancer patients, never the less they felt some things can be done differently as captured in the suggestions below:

*“The medicine is quite strong and the heartbeat changes within five minutes of getting the medication. Now can you imagine at 1:00 pm, at the peak of the sun being told to leave so that others get their treatment? There is no post-chemotherapy resting place for even a few minutes?” (IDI 1)*

*“They need to give us preventive medicines since the complications are known.” (IDI 9)*

#### Improved access to medicines

Participants lamented about the general costs of cancer treatment. Therefore by the time they got oral sores any payment no matter how little, was a strain as illustrated by the comments below:

*“Improve drug availability we can’t afford the costs. I may not be able to get my other cycles since I have run out of money and my sister is also a-day-to-day earner. I have to worry about my health and the money to buy the medicines. I wonder if I won’t die because I can’t buy the medicines.” (IDI 8)*

*“Luckily enough I was admitted so when the doctors came for the round I told them and I was given oracure gel which helped me feed. I would apply it before and after eating. Other medications that were not known to me since my sister was giving me a lot of it for other illnesses as well. Ora-cure was about 21,000 (US$5) I remember it well. But not all can afford it.” (IDI 2)*

## Discussion

One of the key findings of this study was that many patients didn’t get a chance at comprehensive pre-chemotherapeutic counseling and as such were not well-grounded in what to expect as they underwent treatment. This led to a wide array of reactions when mucositis set in. Professional oncology counseling would have allowed for a more balanced and appropriate discussion around chemotherapeutic side effects than that provided by ill-trained cancer patients and or care-givers. In both instances, the expectations of the patients are affected and yet studies have shown that this can have an impact on the severity of symptoms experienced [22]. Therefore it is worthwhile to incorporate training on the management of oral mucositis with clear-cut standard operating procedures for oral hygiene, nutritional/dietary counseling, and pain control for all our nurses at Uganda Cancer Institute. This would increase the knowledgeable pool that can assist the patients.

As expected patients had many side effects that affected them at different levels of severity. From the exchange one could easily tell who had severe mucositis. Those that had severe forms of mucositis struggled quite a bit when it came to feeding and oral hygiene. The struggles varied from total failure to feed and practice oral hygiene to those who only noticed a bit of discomfort. This was in tandem with published findings except that the environment in our study wasn’t as supportive [23]. Rinsing with salty water was a very common aid in cleaning the mouth and although many felt it was inadequate, that is all they could stand. Unfortunately, this is against a background that our patients do not get counseled, assessed, and treated by oral health care personnel before embarking on chemotherapy as is the practice in Europe [23]. Indeed one expressed shock at being turned away when he went for a tooth extraction after revealing that he was under cancer care. Therefore there is a need to avail our patients with the necessary oral health support pre, during, and post-cancer treatment. It would be extremely helpful if oral health care providers became part of the cancer treatment team to avail the necessary pre, during and post oral health care as is the recommended practice [24]. It is worthwhile noting that professional intervention by oral health care providers has been demonstrated to reduce mucositis [25] and as such our patients may benefit from a similar service.

Another clear area that patients pointed out was the need for dietary counseling especially when the mucositis sets in. Since the institute handles many patients from all over the country by now local knowledge on easily available yet comfortable foods should be accessible to the patients. Participants reported a variety of foods that were tolerable with pawpaws (papaya) and watermelon being some of the most tolerable foods. That notwithstanding many preferred drinking during the acute phase of mucositis. Since nearly all patients are treated on an outpatient basis and thus shoulder the responsibility of managing their diet, we should give guidance on what may be more tolerable but highly nutritious as part of the counseling sessions. Although there are recommendations of foods suitable in case of mucositis [26], the stringent financial situation at most homes makes it difficult to get tolerable food items, and as such patients must make do with what the rest of the family eats. This may affect their nutritional status and further complicate treatment. We could borrow a leaf from the early days of highly active antiretroviral therapy (HAART), where food security was shown to affect adherence [27]. The benefits of nutritional counseling have been demonstrated in cancer management [28]. Therefore, some of the the patients lost to follow-up during treatment may be attributable to mucositis and the attendant difficulty in feeding. Food insecurity has been reported for cancer patients in developed countries so the situation is likely to transcend levels of development and thus must be addressed especially in the context of mucositis [29].

Similar to a global perspective as reported by Carlotto et al. [30], our patients were overburdened by the cost of cancer care and this was very common in our study. Many patients because of the excessive treatment financial load complained about the cost of a local anesthetic that is used to soothe pain and allow alimentation. This is against the background that public health care facilities should provide free treatment yet in reality the situation is different [31]. So one can appreciate the mismatch between what is expected and the actual reality. Those who knew the role of leucovorin in oral mucositis avoided treatment unless they had money to get it. Some asked us as to why we cannot have mucositis soothing medications as essential drugs at the cancer institute. Unfortunately, UCI is still struggling with an inconsistent and unpredictable supply of essential chemotherapeutic medications [32]. Additionally, morphine is the recommended pain management medication in case of oral mucositis [33] thus we are less likely to see any of these new mucositis preventive and treatment drugs in the institute pharmacies anytime soon. Although leucovorin’s role in preventing and reducing the severity of mucositis in high dose methotrexate is well established [34], its cost in lieu of the struggles of developing countries like Uganda makes it inaccessible to most and will likely be so for long. However, there are low-cost preventive strategies such as slow infusion and use of ice chips that can lower mucositis incidence and severity [35]. These are within our means and we must strive to bring them on board.

Another area that we need to look at is the potential role of locally available plants that may be of benefit in reducing or shortening the discomfort experienced by those who suffer from mucositis. Our patients reported plants like *Biden’s pilosa*, *Hoslundia opposita* as being helpful but unfortunately, we haven’t tested them to know how effective they could be. Buentzel etal [36] reported several European plants that were effective in treating mucositis but also noted the glaring lag between traditional knowledge and investigative studies when it comes to herbs that may help in alleviating the discomfort from mucositis. A similar situation was noted in China [37] thus we all must improve upon efforts to bring onboard potential locally available herbal remedies through well designed clinical trials This will overcome the methodological challenges that shroud a lot of mucositis intervention research as pointed out by Berger et al. [38].

The results of the present study should be interpreted cautiously because the interviews were run within institute premises and the recruitment team was nurses in the same. Participants were informed that the interviewer was a medical person, which may have affected their reporting of negative comments. There is an opportunity to use online anonymous surveys to get more reliable information as demonstrated by Peach et al. [39]. In future studies, we will look into the viability of this approach in our setting. The participants also took it as an opportunity to air out all their problems that they hadn’t had a chance to address with the treating team making it quite hard to focus on mucositis only. Therefore incorporating patient-related outcomes measures tools in the package we give our patients, would greatly help us interact with our patients and possibly provide solutions to their challenges.

## Conclusion and recommendation

Patients undergoing chemotherapy need to be well prepared before commencing treatment in respect to their oral status, symptoms, and complications. These patients need to have a hotline that allows them access to experienced oncology nursing staff throughout the treatment. This would allow adequate information on side effects and provide quick access to what can be done to alleviate the discomfort associated with mucositis. Additionally, medications that help in relieving pain induced by mucositis should be part and partial of the cancer treatment center essential dugs list to reduce direct out-of-pocket expenditures. Globally we need to run clinical trials on locally available herbs that are used by patients for managing mucositis. This would enable us to advise patients based on evidence generated from their settings.

## Data Availability

Transcripts are available.

## Abbreviations

IDI: In-depth interviews
FGDs: Focus group discussions
UCI: Uganda Cancer Institute
HAART: Highly active antiretroviral therapy
